# Plasminogen in cerebrospinal fluid originates from circulating blood

**DOI:** 10.1186/s12974-014-0154-y

**Published:** 2014-09-16

**Authors:** Anna Mezzapesa, Cyrille Orset, Laurent Plawinski, Loic Doeuvre, Sara Martinez de Lizarrondo, Guglielmina Chimienti, Denis Vivien, Alexandre Mansour, Sabrina Matà, Gabriella Pepe, Eduardo Anglés-Cano

**Affiliations:** Department of Biosciences, Biotechnologies, and Biopharmaceutics, University of Bari, Via Amendola 165/A, 70125 Bari, Italy; Inserm U919, GIP Cyceron, BP 5229, Boulevard Henri Becquerel, 14074 Caen cedex Caen, France; CNRS UMR 5248 CBMN, Institut Européen de Chimie et Biologie, 2 rue Robert Escarpit, 33607 Pessac, France; Department of Neurology, University of Florence, Careggi Hospital, Viale Morgagni 85, 50134 Florence, Italy; Inserm UMRS1140, Faculty of Pharmaceutical and Biological Sciences, Paris Descartes University, 4 Avenue de l’Observatoire, 75270 Paris, cedex 06 France

**Keywords:** Blood–cerebrospinal fluid barrier, Inflammation, LPS, Plasminogen

## Abstract

**Background:**

Plasminogen activation is a ubiquitous source of fibrinolytic and proteolytic activity. Besides its role in prevention of thrombosis, plasminogen is involved in inflammatory reactions in the central nervous system. Plasminogen has been detected in the cerebrospinal fluid (CSF) of patients with inflammatory diseases; however, its origin remains controversial, as the blood–CSF barrier may restrict its diffusion from blood.

**Methods:**

We investigated the origin of plasminogen in CSF using Alexa Fluor 488–labelled rat plasminogen injected into rats with systemic inflammation and blood–CSF barrier dysfunction provoked by lipopolysaccharide (LPS). Near-infrared fluorescence imaging and immunohistochemistry fluorescence microscopy were used to identify plasminogen in brain structures, its concentration and functionality were determined by Western blotting and a chromogenic substrate assay, respectively. In parallel, plasminogen was investigated in CSF from patients with Guillain-Barré syndrome (*n* = 15), multiple sclerosis (*n* = 19) and noninflammatory neurological diseases (*n* = 8).

**Results:**

Endogenous rat plasminogen was detected in higher amounts in the CSF and urine of LPS-treated animals as compared to controls. In LPS-primed rats, circulating Alexa Fluor 488–labelled rat plasminogen was abundantly localized in the choroid plexus, CSF and urine. Plasminogen in human CSF was higher in Guillain-Barré syndrome (median = 1.28 ng/μl (interquartile range (IQR) = 0.66 to 1.59)) as compared to multiple sclerosis (median = 0.3 ng/μl (IQR = 0.16 to 0.61)) and to noninflammatory neurological diseases (median = 0.27 ng/μl (IQR = 0.18 to 0.35)).

**Conclusions:**

Our findings demonstrate that plasminogen is transported from circulating blood into the CSF of rats via the choroid plexus during inflammation. Our data suggest that a similar mechanism may explain the high CSF concentrations of plasminogen detected in patients with inflammation-derived CSF barrier impairment.

**Electronic supplementary material:**

The online version of this article (doi:10.1186/s12974-014-0154-y) contains supplementary material, which is available to authorized users.

## Introduction

Vessel wall fibrinolytic activity and pericellular proteolysis in tissues requires plasminogen binding and transformation into plasmin at the surface of fibrin, cells or the extracellular matrix by either the tissue-type plasminogen activator (tPA) or the urokinase-type plasminogen activator (uPA) [1]. The binding of plasminogen to cells or fibrin is a lysine-dependent mechanism that can be inhibited by lysine analogues such as tranexamic acid (TXA). In the vasculature, plasmin formation results in blood clot dissolution [2], whereas in tissues, plasmin is responsible for pericellular proteolysis accompanying cell migration, angiogenesis, wound healing*,* tissue remodelling and inflammation [3-5]*.* Pericellular proteolysis in the central nervous system (CNS) is involved in development, regeneration of nervous tissues, neuronal and synaptic plasticity and the inflammatory response [6-9]. In humans, plasminogen deficiency is associated with neural disorders including congenital hydrocephalus, periventricular nodular heterotopias and Dandy-Walker malformation [10]. Trace amounts of plasminogen have been reported in normal human cerebrospinal fluid (CSF), whereas raised concentrations have been detected in patients with meningitis [11], subarachnoid haemorrhage [12] and multiple sclerosis [13]*.* However, the question has not been settled as to whether plasminogen in CSF originates from circulating blood or is expressed in the CNS, as suggested by the presence of mRNA in mouse brain [14,15] or by its synthesis by rat microglial cells in culture [16].

The CNS is protected from the bloodstream by the blood–brain barrier (BBB) and the blood–CSF barrier [17,18]. The BBB is the barrier separating the brain interstitial fluid compartment from the general circulation (endothelial tight junctions, basal lamina of endothelial cells and astrocyte pedicles). It selectively limits penetration of a variety of noxious substances and supplies the brain with nutrients. The blood–CSF barrier separates the CSF compartment from blood through choroid plexus epithelial cells, tight junctions, a basal membrane and the endothelium. It restricts the passage of harmful substances from the blood into the CSF secreted across the choroid plexus epithelial cells into the brain’s ventricular system [18]. Because the blood–CSF barrier is more permeable than the BBB, many plasma proteins enter the cerebrospinal liquid (through pinocytosis or active transport). An impairment of the blood–CSF barrier thus leads to an increase in the concentration of proteins in the CSF.

Because diffusion of plasma proteins into the CNS is a selective process, we sought to demonstrate that circulating plasminogen may cross the blood–CSF barrier and can be detected in the CSF. For that purpose, we studied control rats and rats with lipopolysaccharide (LPS)-induced systemic inflammation. Our results show that circulating plasminogen enters the CSF space during blood–CSF barrier dysfunction induced by systemic inflammation, thus suggesting that plasminogen found in the CSF of patients with inflammatory neurological disorders originates from circulating blood.

## Materials and methods

### Reagents and proteins

The enhanced chemiluminescence reagent kit was obtained from Bio-Rad Laboratories (Hercules, CA, USA). The chromogenic substrate selective for plasmin (methylmalonyl)-hydroxyprolylarginine-*para*-nitroaniline (CBS0065) was purchased from Stago (Asnières-sur-Seine, France). *Escherichia coli* LPS serotype 0111:B4, *trans*-4-(aminomethyl)cyclohexane-1-carboxylic acid (or tranexamic acid, TXA), amiloride and 4′,6-diamidino-2-phenylindole (DAPI) and Evans blue were obtained from Sigma Chemical Co (St Louis, MO, USA). Alexa Fluor 488 succinimidyl ester was purchased from Life Technologies (Carlsbad, CA, USA). Goat anti-collagen type IV (Col IV) used for immunohistochemical analyses was obtained from Southern Biotech (Birmingham, AL, USA), and donkey anti-goat antibody F(ab′)2 fragments linked to tetramethylrhodamine isothiocyanate were purchased from Jackson ImmunoResearch (West Grove, PA, USA). Glu-plasminogen, plasmin and peroxidase-conjugated monoclonal antibody against plasminogen kringle 1 (CPL15-PO) were prepared and characterized as described previously [19-21]. A rabbit anti-mouse plasminogen polyclonal antibody was kindly provided by HR Lijnen (University of Leuven, Belgium).

### Patient sampling

A total of 42 patients with neurological diseases attending the Neurological Clinic of the Careggi University Hospital, Florence, Italy, were admitted in this study. Informed consent was obtained according to the Declaration of Helsinki. The Careggi University Hospital Review Board approved the protocol. Diagnoses were based on clinical, laboratory and magnetic resonance imaging data according to the International Classification of Diseases, Tenth Revision, and the *Diagnostic and Statistical Manual of Mental Disorders, Fourth Edition–Text Revision* [22]. Patients with Parkinson’s disease, brain tumour, epilepsy and alcohol or other substance dependence were excluded. All CSF samples were obtained by lumbar puncture and were immediately used for routine laboratory analyses, which included erythrocyte and leucocyte differential cell counts, total protein concentration, albumin and immunoglobulin G (IgG) levels and agarose isoelectric focusing for IgG oligoclonal bands. CSF samples with increased lymphocyte counts (>5 mm^3^) or with blood contamination (erythrocyte concentration >50 cells/mm^3^) were excluded. The CSF to serum albumin quotient (Qalb) was calculated and used to evaluate blood–CSF barrier integrity. Qalb > 0.007 was considered a marker of blood–CSF barrier dysfunction [23,24]. The remainder of each sample was stored in aliquots at −80°C for further analysis.

### Animal experimental model

Experiments were performed using male Wistar rats (280 to 350 g) in accordance with the directives of the Council of the European Communities (86/609/EEC) and the French Agriculture and Forestry Ministry for handling animals (decree 87-848).

#### Purification and labelling of rat plasminogen

To monitor plasminogen uptake by cells and tissues *in vivo* in the rat model of inflammation, we used fluorescence-labelled native plasminogen purified from rat plasma. Blood was obtained by cardiac puncture of anaesthetized animals and collected in 0.129 M sodium citrate. The plasma was separated from blood by centrifugation, and plasminogen was isolated by lysine affinity and sieving chromatography as described previously [19]. The purified plasminogen had a relative molecular weight (M_r_ = 92,000 Da) similar to that of human plasminogen and was labelled with Alexa Fluor 488 dye (A488-Pg) according to the instructions of the manufacturer.

#### Rat model of systemic inflammation

**Figure 1:**
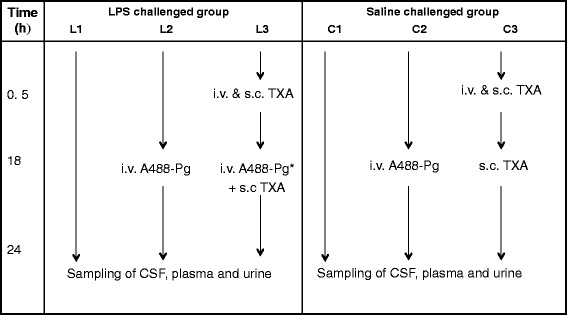
**Diagram of rat inflammatory model and study design.** Rats were separated in six groups (three rats per group). Groups L1, L2 and L3 were challenged with 1 mg/kg lipopolysaccharide (LPS), and the control groups (C1, C2 and C3) were challenged with saline. Both LPS and saline were injected intraperitoneally. *Preincubated with tranexamic acid (TXA); i.v., Intravenous injection of TXA (10 mg/kg) or plasminogen labelled with Alexa Fluor 488 dye (A488-Pg) (1 mg); s.c., Subcutaneous injection of TXA (100 mg/kg).

Rats were assigned to either LPS treatment (L) or the saline control group (C) (*n* = 3 rats per group (Figure 1). Groups L1, L2 and L3 received an intraperitoneal injection of LPS (1 mg/kg) diluted in saline solution, and control groups C1, C2 and C3 received only the saline solution. This dosage of LPS has already been demonstrated to be able to induce brain inflammation in the rat [25]. Groups L1 and C1 received no other treatment. Groups L2 and C2 received a tail vein injection of A488-Pg (1 mg) 18 hours after LPS or saline administration. Group L3 received a tail vein injection of 10 mg/kg TXA 30 minutes after LPS administration, followed by a subcutaneous dose of 100 mg/kg TXA. After 18 hours, each rat received a tail vein injection of A488-Pg (1 mg) preincubated for 15 minutes with 0.1 M TXA, followed by a subcutaneous injection of 100 mg/kg TXA. Group C3 received identical doses of TXA, but not of A488-Pg. This dosage schedule of TXA was necessary to ensure neutralization of lysine-binding sites in both the endogenous and injected plasminogen. At the end of the scheduled treatments (24 hours), samples were collected and animals killed for further studies.

#### Sample collection

The anaesthetized rats were placed in a stereotaxic frame and secured with ear bars, and a midline incision in the skin was made up to the head area to permit easy access to the cisterna magna. The needle, which was connected to a draw syringe, was inserted horizontally and centrally into the cisterna magna for CSF collection. The CSF sample was slowly drawn into the syringe, and the colour of the CSF was closely observed to avoid any possible blood contamination. Approximately 100 μl of CSF was collected from each animal. After a short centrifugation step (3 minutes at 5,000 *g*, 4°C) all CSF samples were immediately flash-frozen in liquid nitrogen and stored at −80°C until use.

Blood was drawn by cardiac puncture using a 1.2 × 40–mm gauge needle syringe containing a 0.1 volume of 0.129 M sodium citrate. Urine was collected by puncturing the bladder. Plasma was separated from blood by centrifugation at 1,500 *g* for 15 minutes. All the samples were kept on ice during collection and kept frozen at −80°C until use.

#### Evans blue permeability assay

Rats (*n* = 3 per group) treated with saline (group C1) or LPS (group L1) alone or supplemented with TXA (groups C3 and L3) received a tail vein injection of Evans blue dye (4% in saline) 4 hours before being killed. After transcardiac perfusion with heparinized saline, the brains and kidneys were harvested and plunged into cold saline until fluorescence measurements were performed. Evans blue dye permeability was monitored by *ex vivo* near-infrared fluorescence (NIRF) imaging using the IVIS 200 imaging system (Caliper Life Sciences, Hopkinton, MA, USA).

#### Fluorescence microscopy

After the rats were killed, parts of the brains were postfixed with 4% paraformaldehyde in 0.1 M phosphate-buffered saline, pH 7.4, at 4°C for 18 hours, followed by 24 hours in 20% sucrose and then frozen in isopentane. For immunocytochemistry, cryostat brain sections (8 μm) were collected on polylysine-coated slides and incubated overnight with a goat anti-Col IV primary antibody (1:1,500), which was revealed with donkey anti-goat antibody F(ab′)2 fragments linked to tetramethylrhodamine isothiocyanate (1:500). The cells were then counterstained with DAPI, mounted with Fluoprep (Dako, Glostrup, Denmark) and observed under an epifluorescence microscope. Images were digitally captured with a Leica DM6000 microscope-coupled CoolSnap camera (Leica Microsystems, Wetzlar, Germany) and visualized with METAVUE 5.0 software (Molecular Devices, Sunnyvale, CA, USA). The detection of exogenously administered A488-Pg in those samples was also studied.

### Detection of functional plasminogen in cerebrospinal fluid

Transformation of CSF plasminogen into plasmin was measured using uPA (5 IU/ml) and a plasmin-selective chromogenic substrate (0.75 mM CBS0065), as previously described [26]. In this system, the initial velocity of *p*-nitroaniline released from CBS0065 is proportional to the amount of plasmin(ogen). The ability of TXA to impair plasminogen binding to the cell membrane was investigated as described previously [27].

### Western blot analysis

Human CSF samples (7 μg of total protein) and rat CSF, urine and plasma (diluted 1:50) samples (10 μl) were electrophoresed in 8% SDS-PAGE gel under nonreducing conditions. Proteins were transferred onto a polyvinylidene fluoride membrane, and plasminogen was revealed using a monoclonal antibody directed against human plasminogen (CPL15-PO, human CSF samples) or a rabbit anti-mouse plasminogen polyclonal antibody (rat samples). The amount of plasminogen was expressed as the number of pixels directly detected using ImageQuant TL 7.0 image analysis software with the ImageQuant LAS 4000 imaging system (GE Healthcare Life Sciences, Pittsburgh, PA, USA) and expressed as nanograms per microlitre by reference to a known amount of plasminogen (15 ng, 10 μl) electrophoresed under similar conditions. In rat samples, the amount of plasminogen was expressed as the number of pixels directly detected using the image analysis software.

### Statistical analysis

Data are representative of at least three independent experiments and are expressed as median (25th to 75th interquartile range (IQR)). The Mann–Whitney *U* test was used to compare values obtained in treated versus control rats. For human experiments, one-way analysis of variance (ANOVA) was performed to compare the patient groups. Bonferroni’s multiple-comparisons posttest was run for the pairwise comparison of groups. Statistical significance was set at *P* < 0.05. A specific statistical package for exact nonparametric inference (Stata Statistical Software release 9 (2005); StataCorp, College Station, TX, USA) was used.

## Results

### Lipopolysaccharide-induced increase in plasminogen in rat cerebrospinal fluid

**Figure 2:**
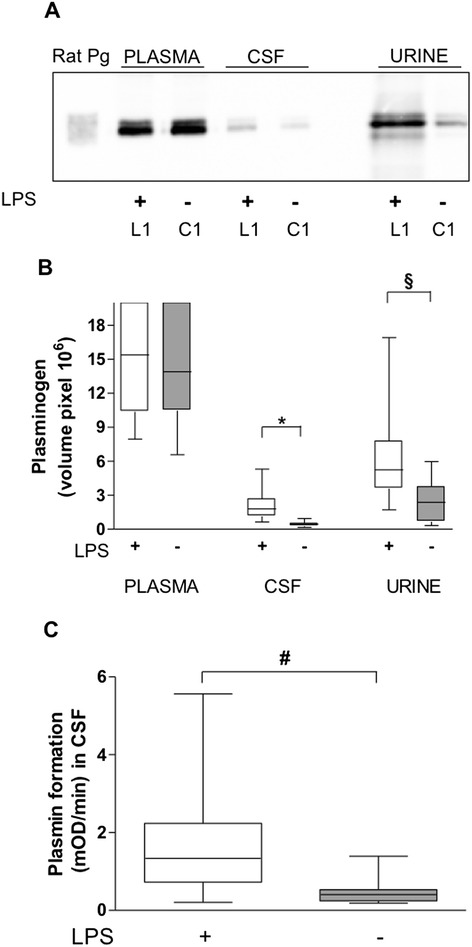
**Determination of plasminogen in rat plasma, cerebrospinal fluid and urine.** Samples were obtained from rats challenged with saline (group C1) versus rats challenged with lipopolysaccharide (LPS) (group L1) (see flowchart in Figure 1). **(A)** Representative immunoblot obtained for plasminogen detection using a rabbit antibody to mouse plasminogen. Electrophoresis of reference rat plasminogen (Pg) in an equal volume (10 μl) of plasma diluted 1:50, cerebrospinal fluid (CSF) and urine. **(B)** Box-and-whisker plot of plasminogen in plasma, CSF and urine after immunoblot and densitometric analyses. Results represent median (25th to 75th interquartile range (IQR)). *P = 0.0002 and §*P* = 0.0071 in CSF and urine (Mann–Whitney *U* test), respectively. **(C)** Box-and-whisker plot of measurement of the activation of plasminogen in CSF samples (chromogenic substrate assay). Results represent median (IQR) of the velocity of plasmin formation. #*P* = 0.0045 (Mann–Whitney *U* test). mOD: milli optical density.

Endogenous plasminogen was detected by Western immunoblotting in plasma, CSF and urine of both LPS- and saline-treated control rats (groups L1 and C1, respectively) (Figure 2A). The LPS injection produced no significant variation in the amount of circulating plasminogen as compared to controls (0.1-fold increase in injected versus control rats) (Figure 2B). In contrast, we detected higher plasminogen levels in CSF and urine from LPS-treated rats (fourfold increase in CSF, *P =* 0.0002; twofold increase in urine, *P* = 0.0071 (both by Mann–Whitney *U* test)) (Figure 2B). Rat CSF plasminogen was efficiently transformed into plasmin; its active concentration appeared to be directly related to the amount of plasminogen detected in CSF samples by Western immunoblotting (*P* = 0.0045, Mann–Whitney *U* test) (Figure 2C).

### Lipopolysaccharide-induced increase in barrier permeability

**Figure 3:**
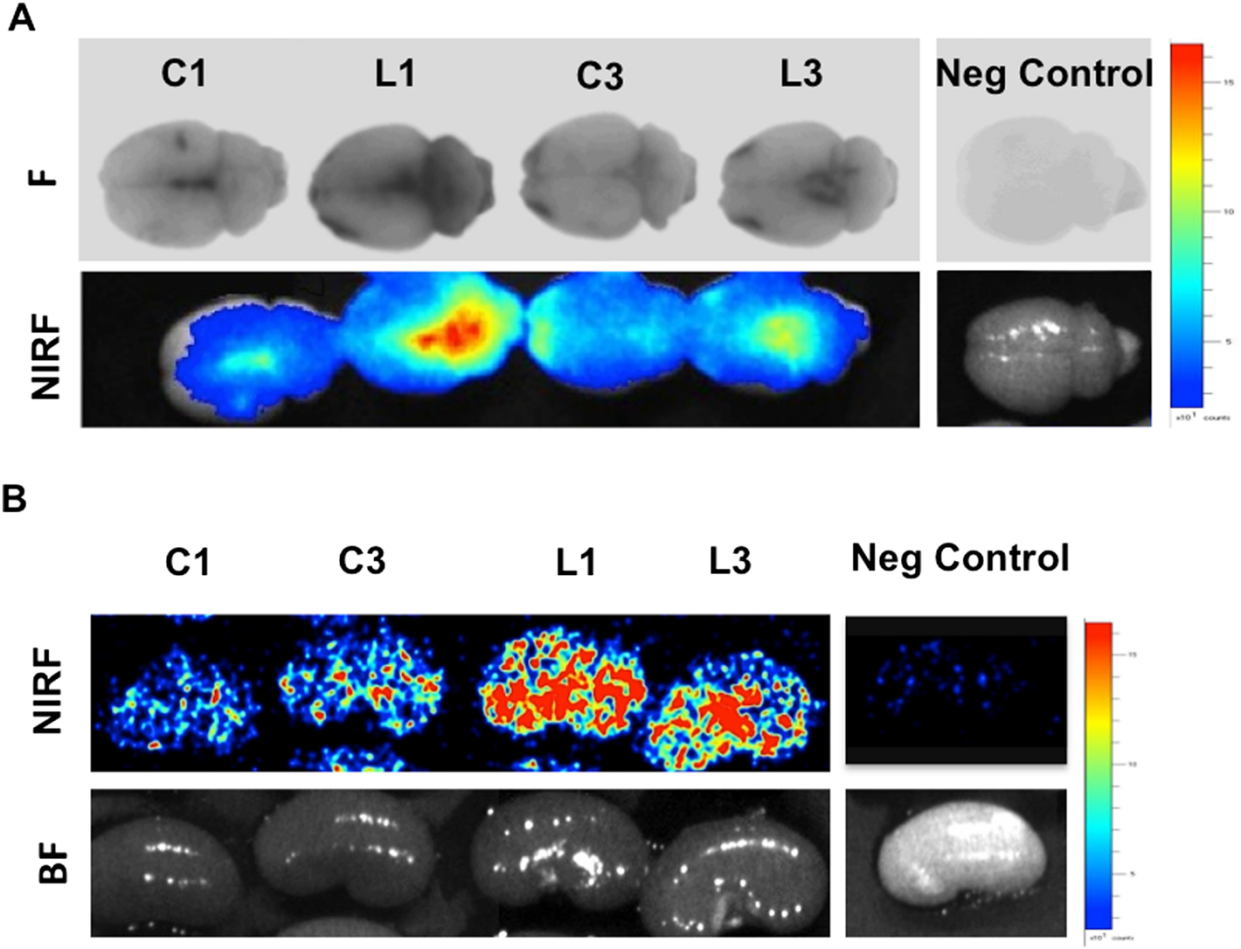
**Blood–cerebrospinal fluid barrier and glomerular barrier permeability to Evans blue dye.** *Ex vivo* fluorescent (F) and near-infrared fluorescent (NIRF) images of brains and kidneys from rats injected with Evans blue dye 24 hours after lipopolysaccharide (LPS) treatment according to the outline in Figure 1. The measurement was performed in perfused brains 24 hours after Evans blue dye injection. C1, Saline-challenged rats; L1, LPS-challenged rats; C3, Saline/tranexamic acid (TXA); L3, LPS/TXA; Neg control, Rat without Evans blue dye; BF, Bright field. **(A)** Brain NIRF images. **(B)** Kidney NIRF images. The high fluorescence intensity observed in LPS-treated rats (L1 and L3) compared to saline-treated rats (C1 and C3) indicates leakage of the blood–cerebrospinal fluid barrier (A) or the glomerular filtration barrier (B). No differences were observed between saline-treated (C1) and TXA-treated (C3) animals, indicating no effect of TXA on extravasation of Evans blue dye.

The aforementioned data suggest that vascular permeability was modified following the LPS injection. This hypothesis was investigated by testing permeability to Evans blue dye. *Ex vivo* fluorescent and NIRF images showed higher brain fluorescence in the LPS-treated rats (group L) as compared to controls (group C), indicating an increase in barrier permeability to the dye due to blood–CSF barrier impairment (Figure 3A). Modifications in the glomerular filtration barrier were also detected with the Evans blue test (Figure 3B). Higher kidney fluorescence was observed in LPS-treated rats (group L) as compared to controls (group C). The administration of TXA (groups C3 and L3) was without effect on barrier permeability.

### *In vivo* evidence of the circulating origin of plasminogen in cerebrospinal fluid

**Figure 4:**
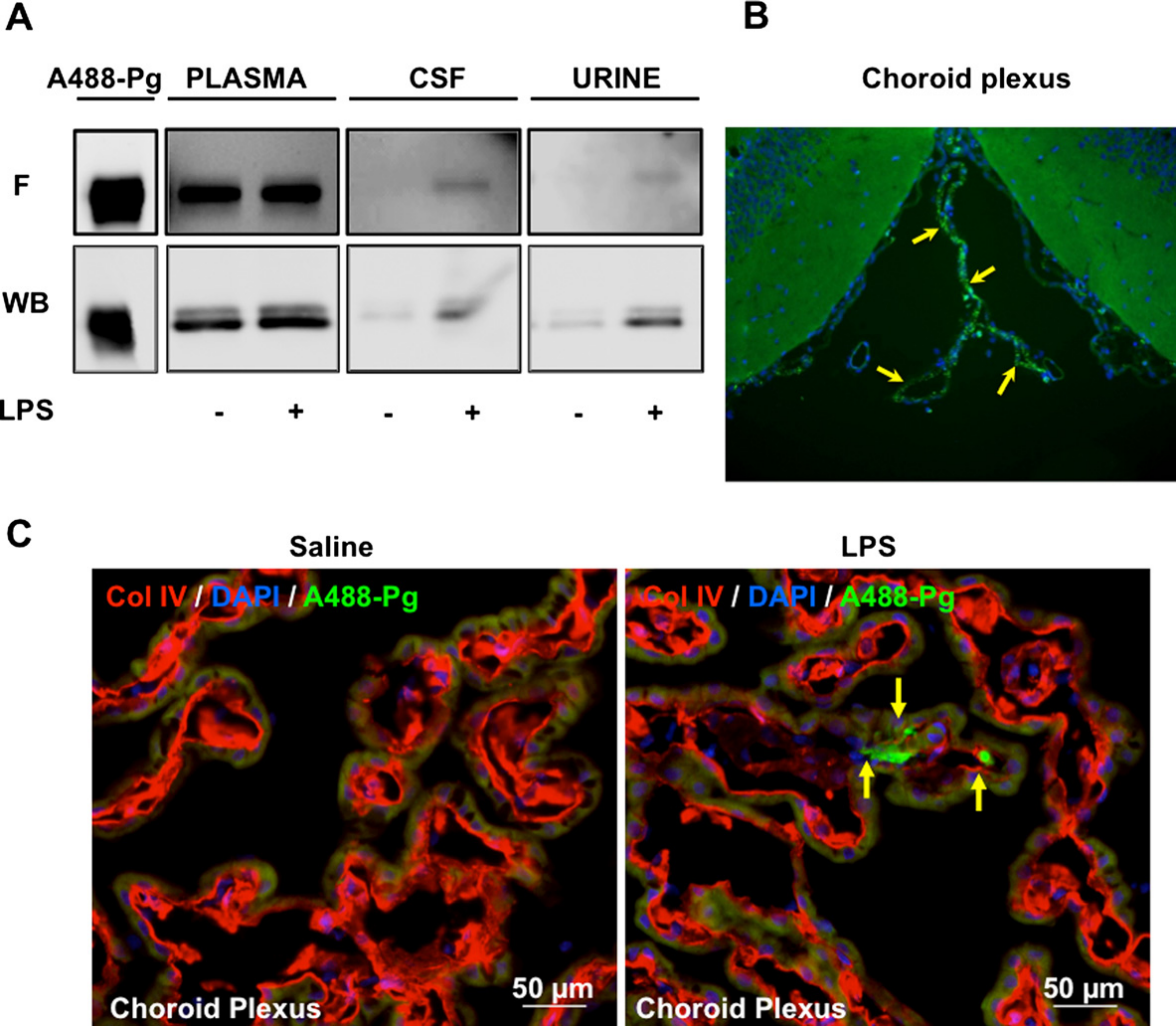
***In vivo*** **evidence of the circulating origin of plasminogen in cerebrospinal fluid.** Samples were obtained from rats injected with plasminogen labelled with Alexa Fluor 488 dye (A488-Pg). Group L2 rats were challenged with lipopolysaccharide (LPS), and group C2 was challenged with saline (see flowchart in Figure 1). **(A)**. An equal volume (10 μl) of either plasma diluted 1:50 or of cerebrospinal fluid (CSF) or urine was electrophoresed, and fluorescence (F) in the gel was directly revealed using ImageQuant TL 7.0 image analysis software (upper panel). The gel was then transferred onto a polyvinylidene fluoride membrane and detected by Western blotting with a rabbit antibody to mouse plasminogen (WB, lower panel). Representative samples are shown. **(B)** Micrograph showing the presence of circulating A488-Pg (indicated by arrows) in the choroid plexus of an LPS-treated rats detected by direct fluorescence microscopy. 4′,6-diamidino-2-phenylindole (DAPI) staining (blue) indicates cell nuclei. **(C)** Magnified images of choroid plexus of saline- and LPS-treated rats showing the presence of circulating A488-Pg (indicated by yellow arrows) only in the LPS condition, as detected by direct fluorescence microscopy. DAPI staining (blue) indicates cell nuclei, and collagen type IV (Col IV, red) is used as a vessel marker.

To obtain *in vivo* evidence of the extravasation of plasminogen from blood, animals were challenged with exogenous A488-Pg 18 hours after the LPS (group L2) or saline injection (group C2). A488-Pg appeared in the CSF and urine of all LPS-treated rats, indicating that blood plasminogen crosses the barrier during its presence in the circulation (with a half-life of about 2 days) (Figure 4A) and integrates the CSF exchanged at least three times daily [28]. Actually, the presence of circulating A488-Pg was clearly visualized by fluorescence microscopy only in the choroid plexus of LPS-treated rats (Figure 4B). Figure 4C is a magnified view of the choroid plexus showing the presence of A488-Pg and the vessel wall detected by immunocytochemistry of Col IV. Of note, A488-Pg is absent in the saline-treated control.

**Figure 5:**
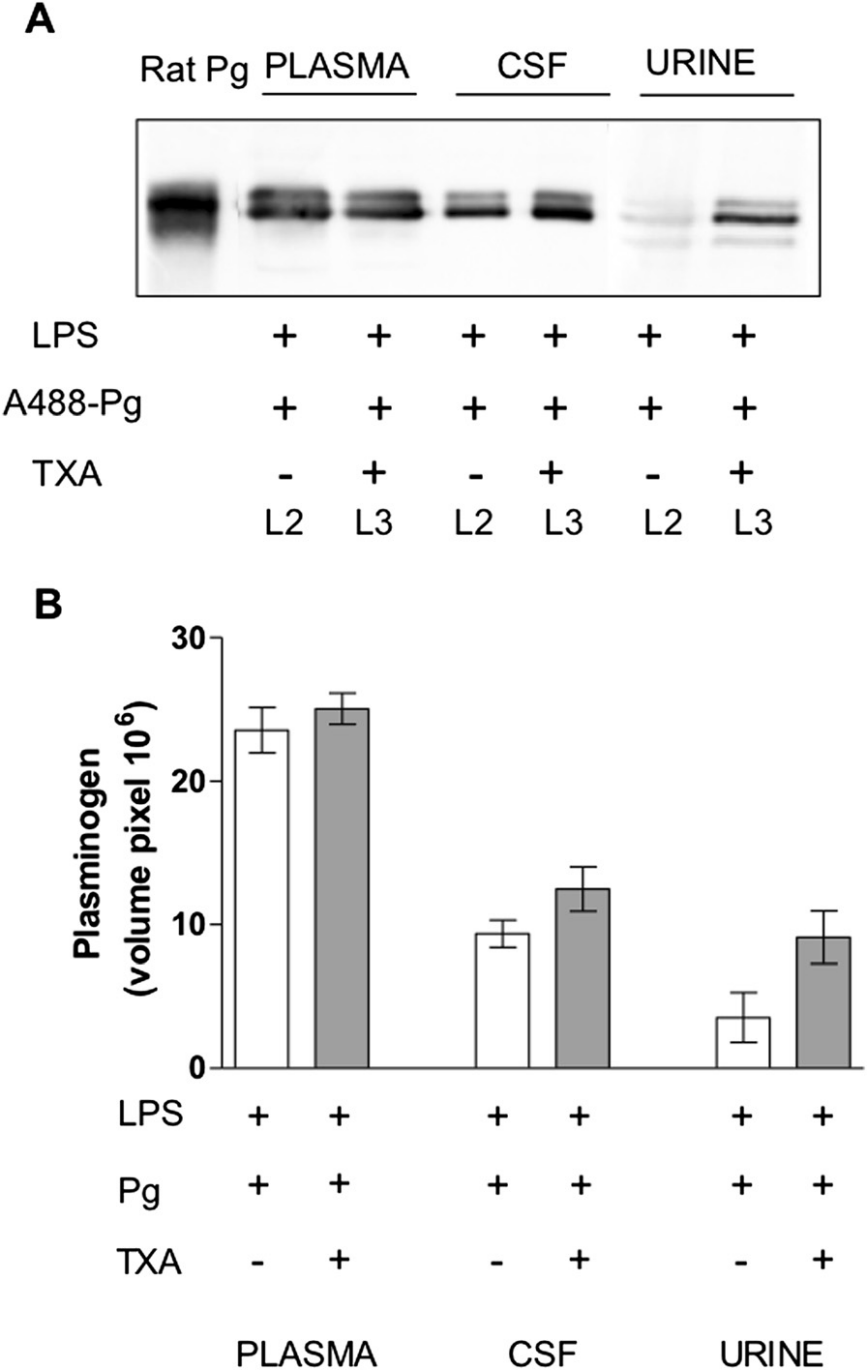
**Effect of tranexamic acid on plasminogen transfer through the blood–cerebrospinal fluid barrier and the renal glomerulus.** Rats with lipopolysaccharide (LPS)-induced systemic inflammation received an intravenous injection of plasminogen labelled with Alexa Fluor 488 dye (A488-Pg) alone (group L2) or in the presence of sustained concentrations of tranexamic acid (TXA) (group L3) (see flowchart in Figure 1). Plasminogen was detected by immunoblotting. **(A)** Representative immunoblot of rat plasminogen (Pg), plasma diluted 1:50 cerebrospinal fluid (CSF) and urine samples using a rabbit antibody to mouse plasminogen. Experiments were performed as indicated in Figure 2 with an equal volume (10 μl) of each sample. **(B)** Representative column bars of plasminogen in plasma, CSF and urine after immunoblot and densitometric analysis.

Typically, binding of plasminogen by cells is mediated by a lysine-dependent mechanism via lysine residues encompassed in cellular receptors and lysine-binding sites located in plasminogen kringle domains [1]. To explore whether the passage of plasminogen through the blood–CSF barrier implicates a lysine-dependent mechanism, we measured cellular uptake of A488-Pg in the presence of the lysine analogue TXA. Figure 5 shows that plasminogen remained increased in the CSF of rats treated with TXA, thus excluding uptake via known plasminogen receptors harbouring carboxy-terminal lysine residues [1].

**Figure 6:**
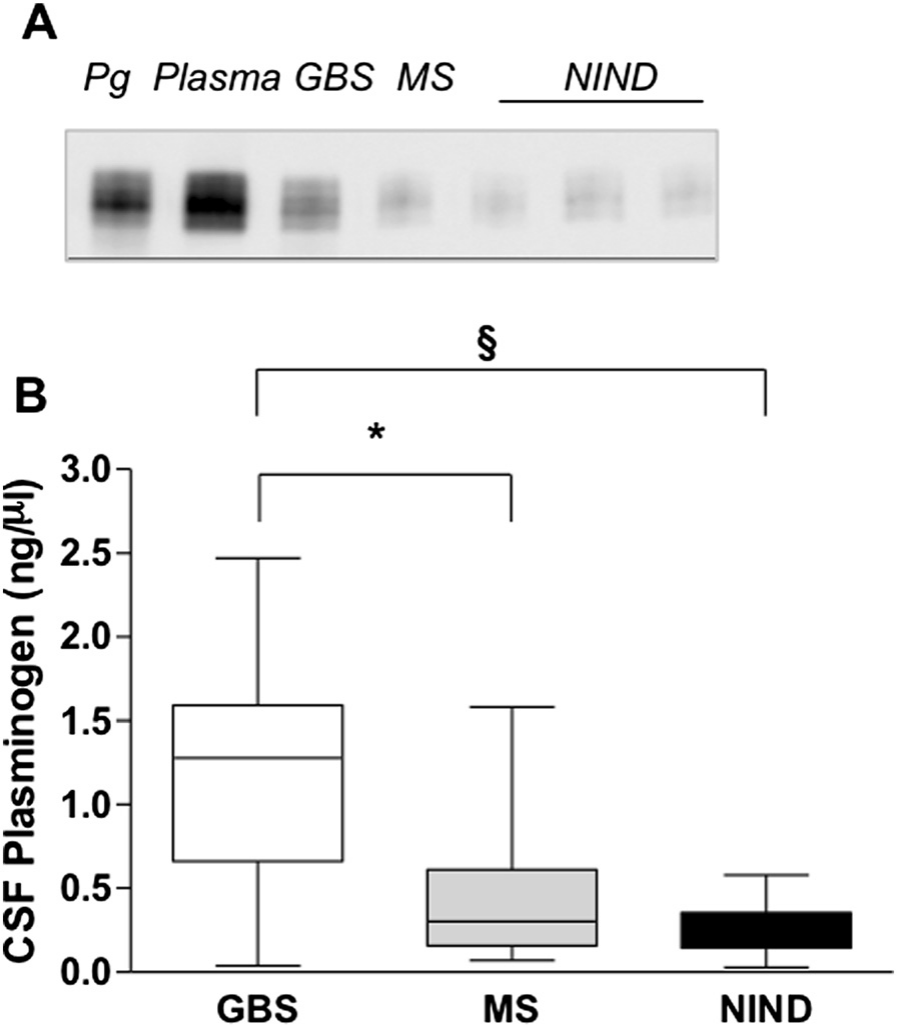
**Quantification of plasminogen in human cerebrospinal fluid.** Cerebrospinal fluid (CSF) samples were collected from patients with Guillain-Barré syndrome (GBS, *n* = 15), multiple sclerosis (MS, *n* = 19) and noninflammatory neurological disease (NIND, *n* = 8). **(A)** Representative human CSF immunoblot showing peroxidase-conjugated monoclonal antibody directed against plasminogen kringle 1 (CPL15-PO). Electrophoresis of human plasminogen (Pg) (M_r_ = 92,000 Da), 10 μl of human plasma diluted 1:50 and 7 μg of total protein in CSF. **(B)** Box-and-whisker plot of human CSF plasminogen after immunoblot analysis and densitometric analysis. Results represent median (interquartile range) (*P* < 0.0001, one-way analysis of variance). *P < 0.05 for GBS versus MS and §*P* < 0.05 for GBS versus NIND (Bonferroni’s multiple-comparisons posttest).

To substantiate the relevance of these *in vivo* rat studies to human pathology, we investigated the presence of plasminogen in the CSF of patients with inflammatory and noninflammatory disorders. Plasminogen antigen was detected in all CSF human samples (Figure 6). Higher concentrations of plasminogen were found in Guillain-Barré syndrome (GBS) (median = 1.28 ng/μl (25th to 75th interquartile range (IQR) = 0.66 to 1.59), *n* = 15) as compared to multiple sclerosis (MS) (median = 0.3 ng/μl (IQR = 0.16 to 0.61), *n* = 19) and noninflammatory neurological diseases (NINDs) (median = 0.27 ng/μl (IQR = 0.18 to 0.35), *n* = 8) (*P <* 0.0001, one-way ANOVA). The pairwise comparison yielded significant differences in GBS versus MS (*P* < 0.05) and GBS versus NINDs (*P* < 0.05), both analysed with Bonferroni’s posttest. GBS patients had significantly higher values of CSF total protein, albumin and IgG, as well as higher Qalb, whereas MS patients had higher index values of IgG (QIgG/Qalb) (see Additional file 1: Table S1).

## Discussion

Plasminogen is synthesized mainly by the liver and then is distributed to tissues via the systemic circulation [29]. The concentration of plasminogen in circulating blood is relatively high (1.5 to 2 μM) and constant, as plasminogen does not behave as an acute phase reactant such as fibrinogen. Therefore, inflammatory conditions or the injection of an inflammatory agent such as LPS do not increase the circulating concentration of plasminogen. However, a mechanistic link between inflammation and the blood–CSF barrier dysfunction has been established [30], which may explain an increased transfer of plasminogen from circulating blood to CSF. Indeed, trace amounts of plasminogen have been found in the CSF of patients with noninflammatory diseases [31,32]. By using an LPS-induced model of systemic inflammation in rats, we found that plasminogen is present at significantly higher concentrations in the CSF of LPS-treated rats as compared to controls. Accordingly, LPS-treated rats had increased blood–CSF barrier permeability, as demonstrated with Evans blue dye. CSF plasminogen is efficiently transformed into plasmin, thus suggesting that transfer of plasminogen from circulating blood to the CSF could be a source for plasmin formation in the nervous system. Furthermore, using A488-Pg, we found that this labelled exogenous rat plasminogen crosses the blood–CSF barrier. The half-life of plasminogen in the circulation is about 2 days, whereas the CSF is produced and exchanged by cells of the choroid plexus at least three times daily [28]. Thus, circulating A488-Pg could be detected in the CSF and the choroid plexus of LPS-treated rats. These *in vivo* data suggest that circulating plasminogen enters the CSF at the choroid plexus. This enormous epithelial/endothelial surface area is potentially available for untoward leakage of plasma proteins into CSF [33,34]. Because LPS is also known to induce interference with the integrity of the glomerular filtration barrier [35], we also investigated the passage of plasminogen through the renal glomerulus. We detected modifications in the glomerular filtration barrier with the Evans blue test and high concentrations of both endogenous plasminogen and A488-Pg in the urine of LPS-treated rats as compared to trace amounts found in control rats.

Binding of plasminogen to carboxy-terminal lysine residues of membrane receptors is a well-known mechanism governing its capture and concentration onto the cell surface, where it can be either transformed into plasmin or internalized [1]. Because these receptors could potentially be involved in the transfer of plasminogen from blood to CSF, we tested the possibility that TXA, a lysine analogue, might prevent plasminogen from entering the CSF. Occupation of the lysine-binding site of plasminogen by TXA prevents binding to lysine residues of membrane glycoprotein receptors. However, TXA did not inhibit A488-Pg transfer through the blood–CSF barrier, thus suggesting that other binding sites might be involved in plasminogen uptake. For instance, a peptide derived from the plasminogen-binding site domain 1 of M6PIGF2R, peptide fragment 18 to 36, induces plasminogen internalization [36].

Collectively, these findings demonstrate that, during inflammation, plasminogen is transported from circulating blood into the CSF of rats via the choroid plexus. Interestingly, the high plasminogen CSF content detected in LPS-treated versus control rats parallels the significantly higher concentration of plasminogen found in the CSF of patients with blood–CSF barrier dysfunction determined on the basis of a high Qalb. In agreement with previously published data [31,32], we found that plasminogen is present in trace amounts in noninflammatory CSF samples. However, we found significantly higher concentrations in the CSF of patients with blood–CSF barrier dysfunction. Our present data are in accord with our previous demonstration of the presence of apolipoprotein(a), a glycoprotein homologous to plasminogen in the CSF of patients with blood–CSF barrier dysfunction [37].

Although there are limitations to translating results obtained in experimental animals to human diseases, our data are suggestive of a similar mechanism for plasminogen transfer from blood to CSF and may be of relevance to patients with inflammation-derived CSF barrier impairment.

## Conclusions

We have demonstrated that plasminogen is increased in the CSF of patients with a dysfunctional blood–CSF barrier. We have reproduced, in an *in vivo* rat model of systemic inflammation, similar modifications of barrier function, and we also have demonstrated that plasminogen from circulating blood enters the CSF space. Altogether our data strongly suggest that the increase in CSF plasminogen concentrations detected in patients obeys a mechanism by which circulating plasminogen crosses the altered blood–CSF barrier.

## Additional file

Additional file 1: Table S1. Study groups and CSF biochemical parameters.

## Abbreviations

A488-Pg: Alexa Fluor 488 plasminogen
CBS0065: (methylmalonyl)-hydroxyprolylarginine-*para*-nitroaniline
CNS: Central nervous system
CSF: Cerebrospinal fluid
DAPI: 4′,6-diamidino-2-phenylindole
GBS: Guillain-Barré syndrome
MS: Multiple sclerosis
NIND: Noninflammatory neurological disease
NIRF: Near-infrared fluorescence
TXA: Tranexamic acid
uPA: Urokinase-type plasminogen activator
