# Supplementary material for: Plasminogen in cerebrospinal fluid originates from circulating blood
Source: J Neuroinflammation. 2014 Sep 16;11:154. doi: 10.1186/s12974-014-0154-y (PMC4173110; doi:10.1186/s12974-014-0154-y)
Supplement: Additional file 1: Table S1. — Study groups and CSF biochemical parameters. [file 12974_2014_154_MOESM1_ESM.docx]

**Additional file 1: Table S1. Study groups and CSF biochemical parameters**

| **Subjects** | **GBS (n. 15)** | **MS (n. 19)** | **NIND (n. 8)** |
| --- | --- | --- | --- |
| **Female/male** | 7/8 | 7/12 | 4/4 |
| **Age** | 39.0 (18-80) | 37.0 (20-60) | 49.5 (18-68) |
| **CSF protein (µg/µl)** | 1.23 (0.96-1.9) | 0.49 (0.33-0.55) | 0.35 (0.24-0.61) |
| **CSF albumin (µg/ml)** | 96.0 (73.9-144) | 35.0 (20.7-40) | 23.7 (17.2-43.7) |
| **CSF IgG**  **(µg/ml)** | 19.8 (12.3-30.7) | 5.1 (4.1-6.6) | 3.5 (2.8-5.2) |
| **Qalb x 100** | 2 (1.7-4) | 0.8 (0.5-1) | 0.5 (0.4-0.1) |
| **QIgG x 100** | 1.9 (0.9-3.2) | 0.5 (0.4-0.7) | 0.3 (0.2-0.6) |
| **Link Index** | 0.69 (0.6-0.8) | 0.6 (0.49-0.88) | 0.5 (0.45-0.55) |

Sex distribution, median age (range). CSF protein concentration in patients with Guillan-Barré syndrome (GBS), multiple sclerosis (MS) and non-inflammatory neurological diseases (NIND).

CSF: cerebrospinal fluid; Qalb:CSF/serum albumin ratio; QIgG: CSF/serum IgG ratio; Link Index: (QIgG/Qalb). All values are medians (25^th^ to 75^th^ interquartile range).
